# Supplementary material for: Gene expression drives the evolution of dominance
Source: Nat Commun. 2018 Jul 16;9:2750. doi: 10.1038/s41467-018-05281-7 (PMC6048131; doi:10.1038/s41467-018-05281-7)
Supplement: Supplementary file 1 — Supplementary Information [file 41467_2018_5281_MOESM1_ESM.pdf]

Supplementary Information

Gene expression drives the evolution of dominance

Huber *et al.*

1. Supplementary Figures 1-13
2. Supplementary Tables 1-5
3. Supplementary Note 1
4. Supplementary References

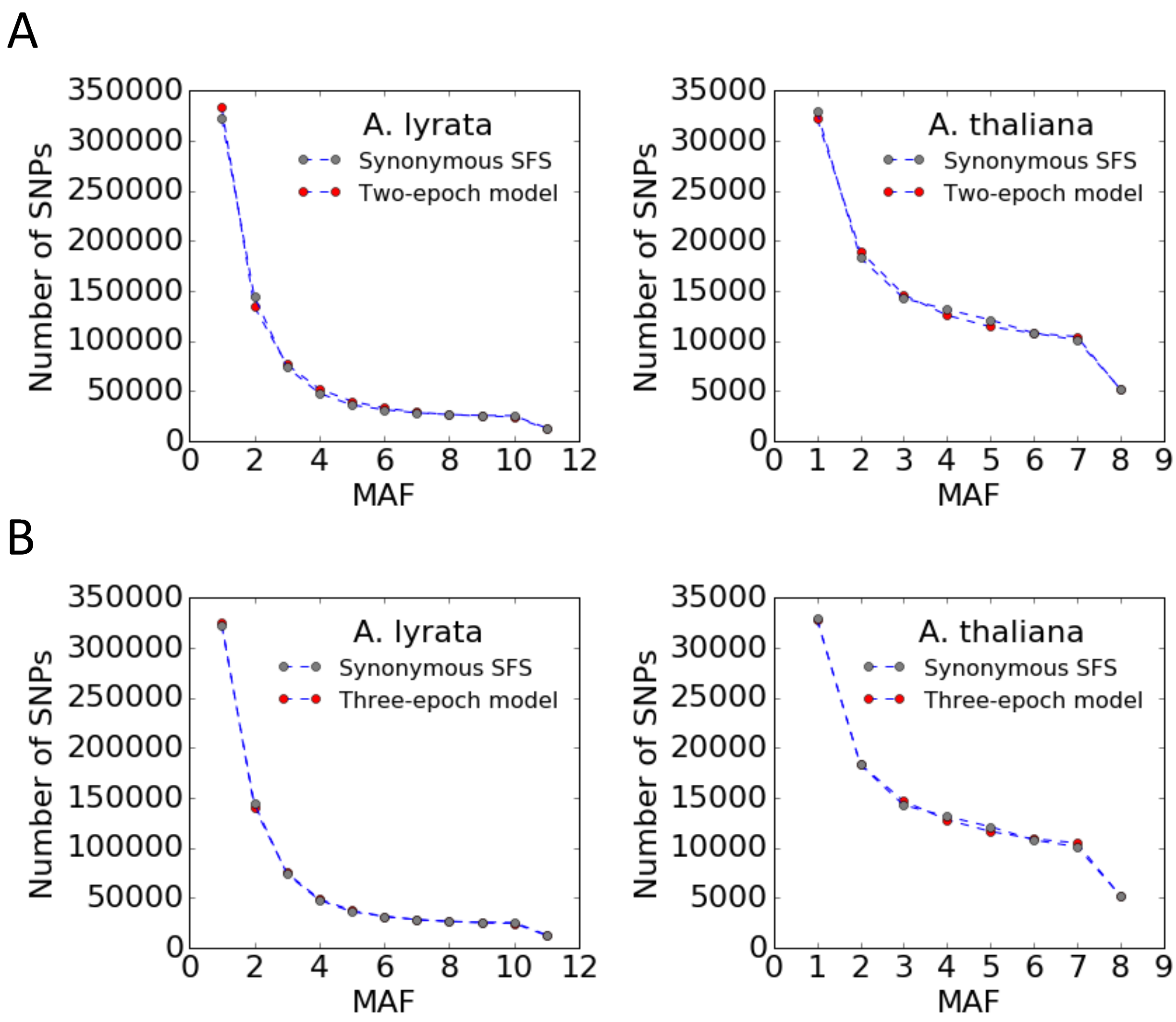

Supplementary Figure 1. Demographic model fit to the synonymous SFS.
MAF is the minor allele frequency. In both species, the three-epoch model (B) fits singletons and doubletons better than the two-epoch model (A).

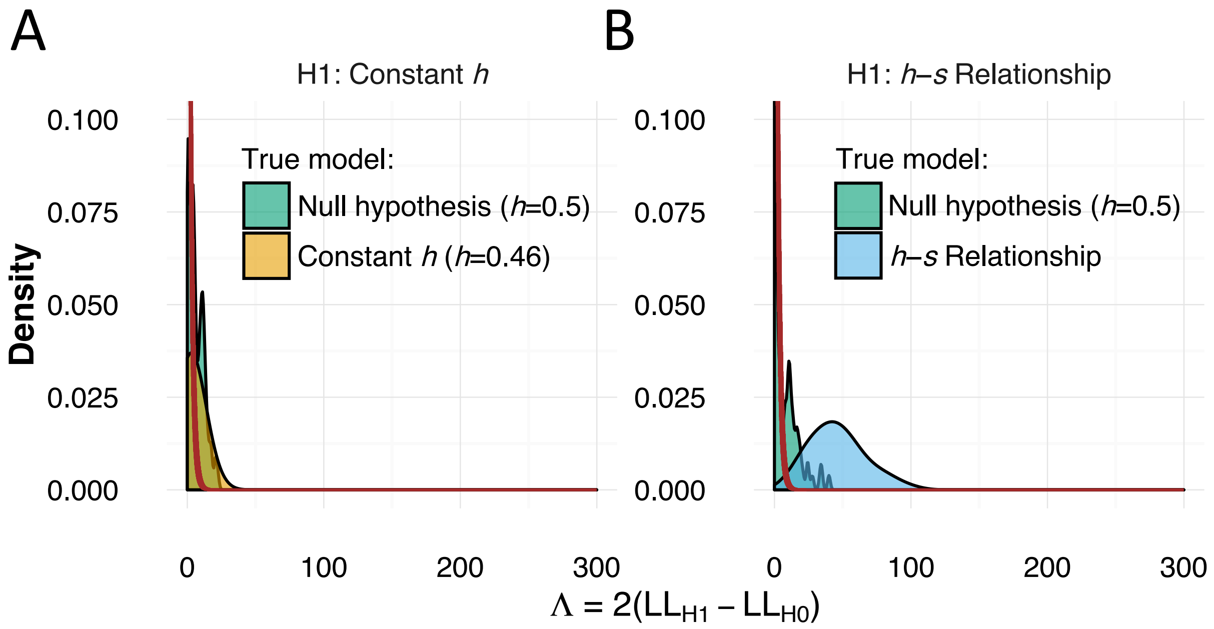

Supplementary Figure 2. Power for discriminating between dominance models using data from a single outcrossing species (*A. lyrata*).
(A) Likelihood ratio tests comparing a constant *h* model to an additive model. When data are simulated under an additive model (green), Λ nearly follows a chi-square (2 *df*) distribution (red line). When the data are simulated under a model with *h*=0.46 (tan), the distribution of Λ overlaps considerably, indicating little statistical power. (B) Likelihood ratio tests comparing the *h-s* relationship model to an additive model. When data are simulated under an additive model (green), Λ nearly follows a chi-square (2 *df*) distribution (red line). However, when the data are simulated under the *h-s* relationship model (blue), the distribution of Λ is substantially larger, indicating good statistical power.

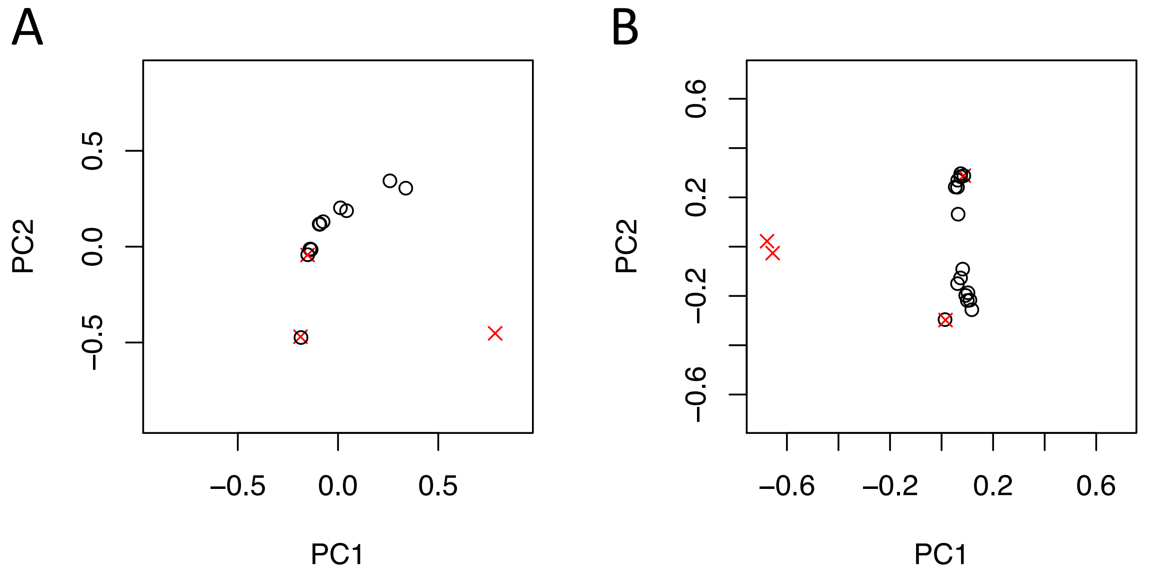

Supplementary Figure 3. Principal component analysis of population structure. Principal component analysis (PCA) of the genetic structure of (A) *A. lyrata* and (B) *A. thaliana*. When two accessions were closely related, we retained one individual selected at random. We also removed accessions that are highly diverged from the majority of individuals. The accessions that we removed are indicated by red crosses.

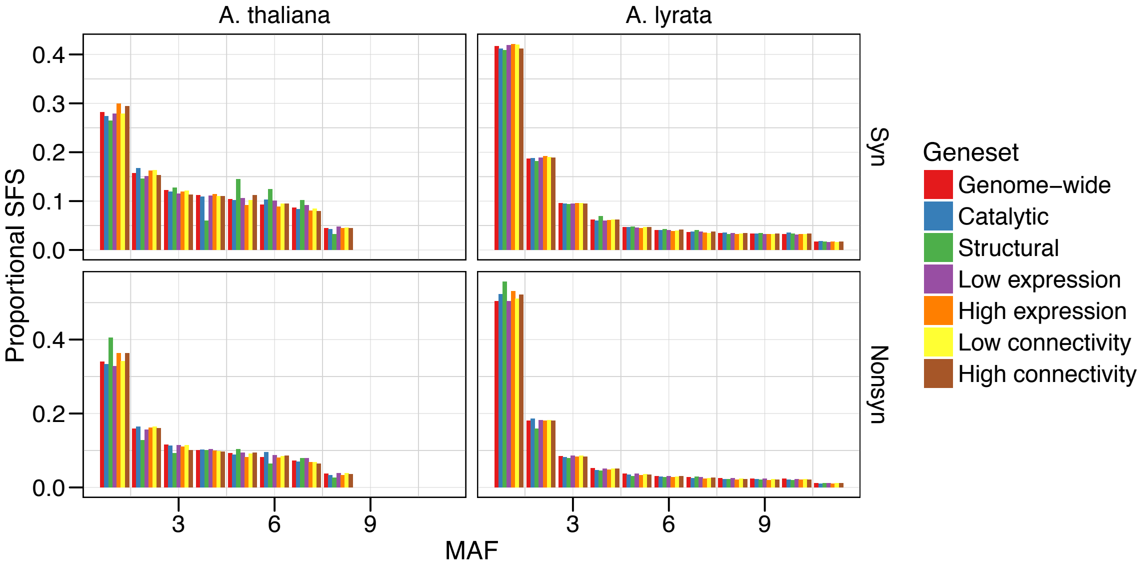

Supplementary Figure 4. Folded site frequency spectra (SFS) for different categories of genes.
In both species, structural proteins have the highest proportion of nonsynonymous singletons, suggesting these genes have experienced a greater effect of purifying selection.

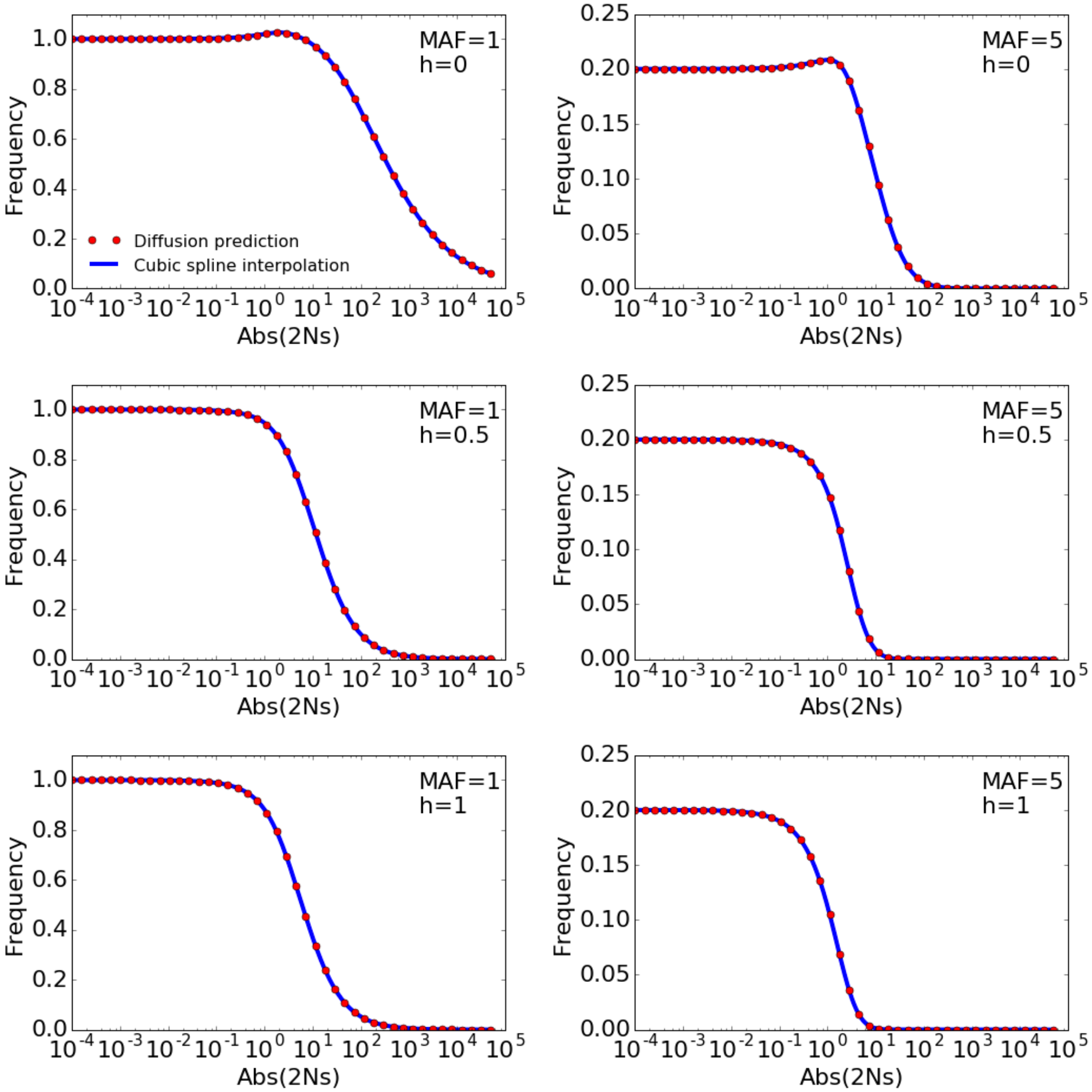

Supplementary Figure 5. Cubic spline interpolation of the SFS along the $N_e s$ axis. Examples of cubic spline interpolation of two entries of the SFS (MAF=1 and MAF=5) for *h*=0, *h*=0.5, and *h*=1. The blue line is the cubic spline interpolation to the red points, which indicate the expected values under the diffusion approximation as predicted by ∂a∂i. The demography is assumed to be a constant size model. In all cases, the interpolation line fits well to the red points.

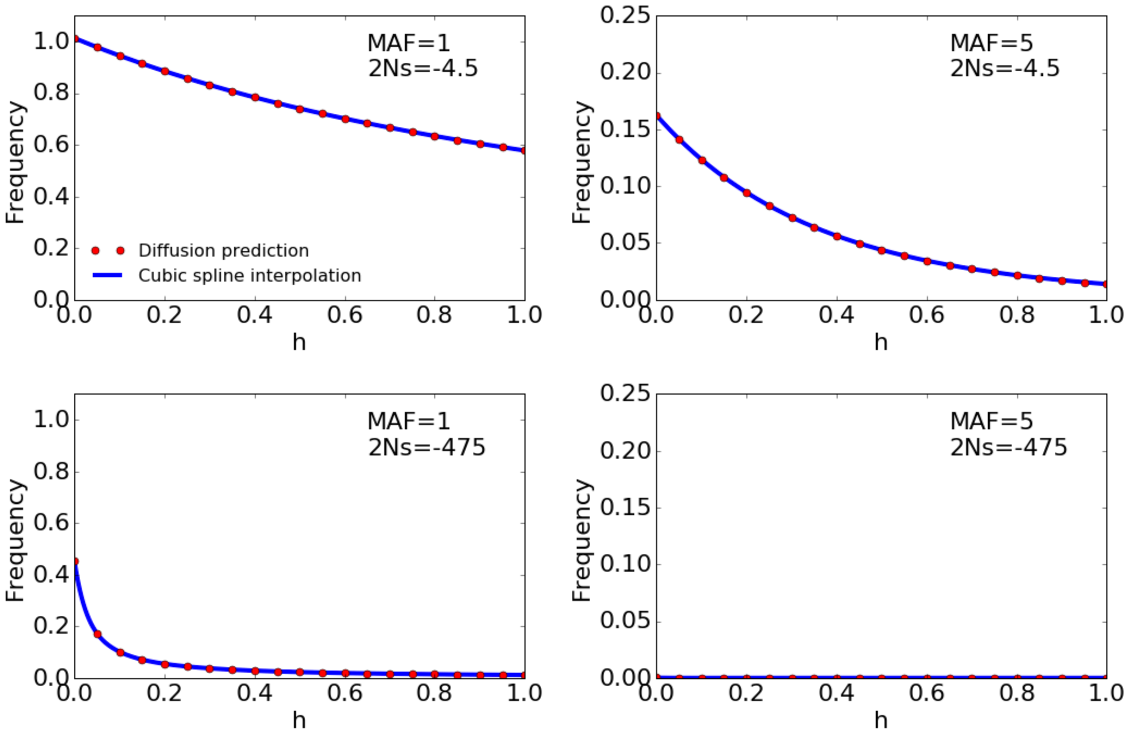

Supplementary Figure 6. Cubic spline interpolation of the SFS along the *h* axis. Examples of cubic spline interpolation of two entries of the SFS (MAF=1 and MAF=5) for slightly deleterious (2*Ns=-4.5)* and strongly deleterious (2*Ns*=-475) mutations. The blue line is the cubic spline interpolation to the red points, which indicate the expected values under the diffusion approximation as predicted by ∂a∂i. The demography is assumed to be a constant size model. In all cases, the interpolation line fits well to the red points.

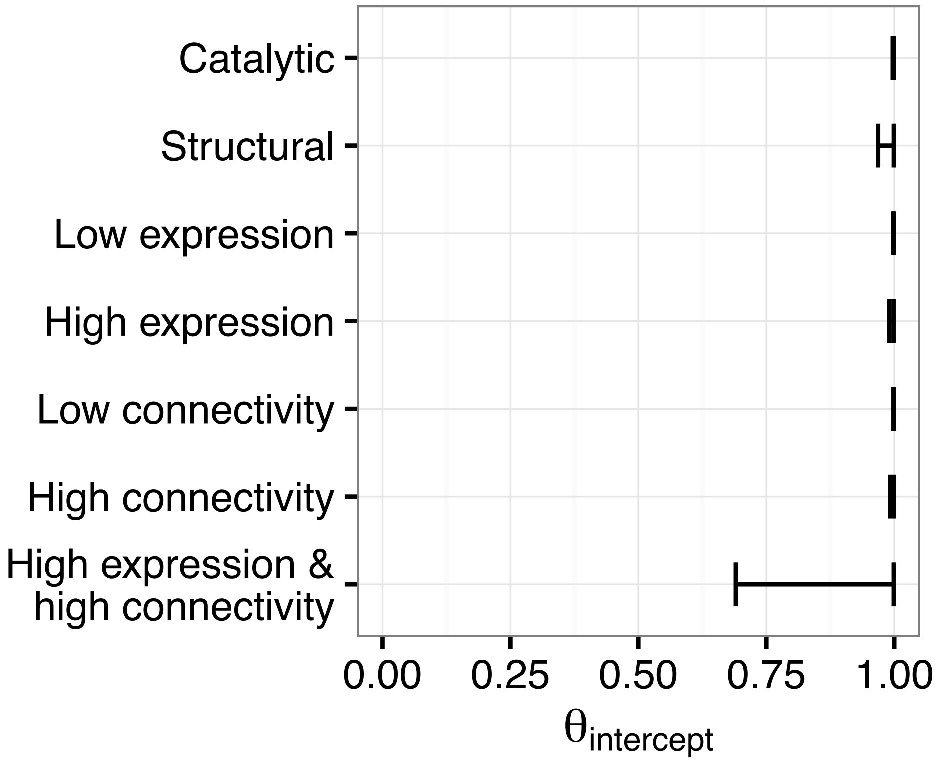

Supplementary Figure 7. Estimation of the intercept parameter of the *h-s* relationship. Confidence interval (95%) for the estimate of $\theta_{intercept}$ for different gene categories, combining data from *A. lyrata* and *A. thaliana* for estimation. Note that the confidence intervals for $\theta_{intercept}$ for different categories of genes overlap each other suggesting no difference in this parameter.

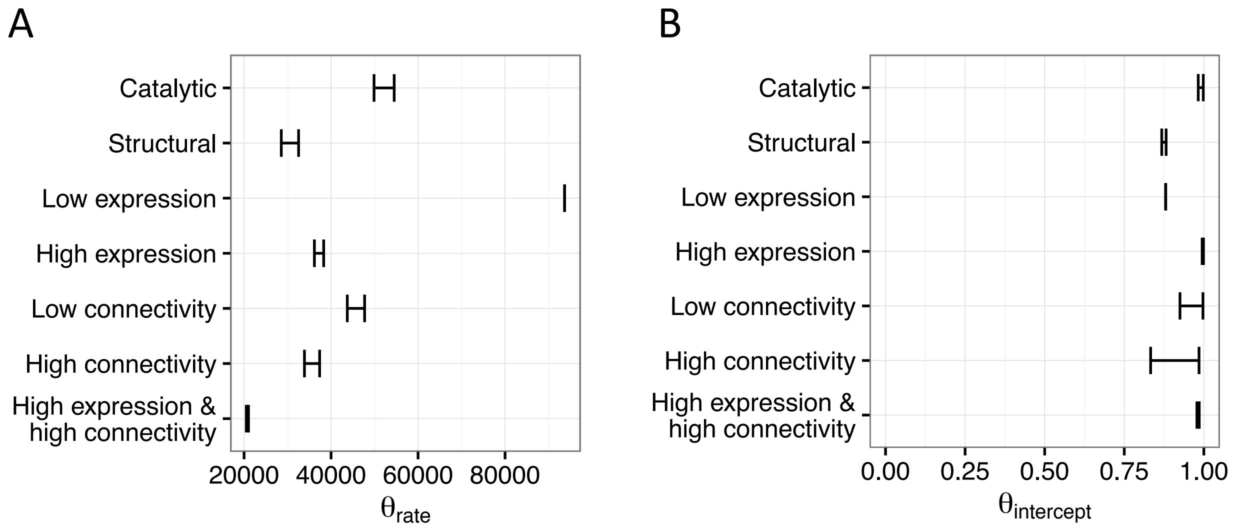

Supplementary Figure 8. Estimation of the intercept and rate parameter of the *h-s* relationship.
Confidence interval (95%) for the estimate of θ_{rate} (A), and $\theta_{intercept}$ (B), for different gene categories using only data from *A. lyrata* for estimation. Similar to Fig. 4c, the structural genes as well as the high expression & high connectivity genes show the smallest θ_{rate} estimate, whereas catalytic genes, low expression genes, and low connectivity genes show the highest θ_{rate} estimate. However, the confidence intervals are in general larger and more variable between gene sets than when estimating the parameters using both *A. lyrata* and *A. thaliana* (Fig. 4c).

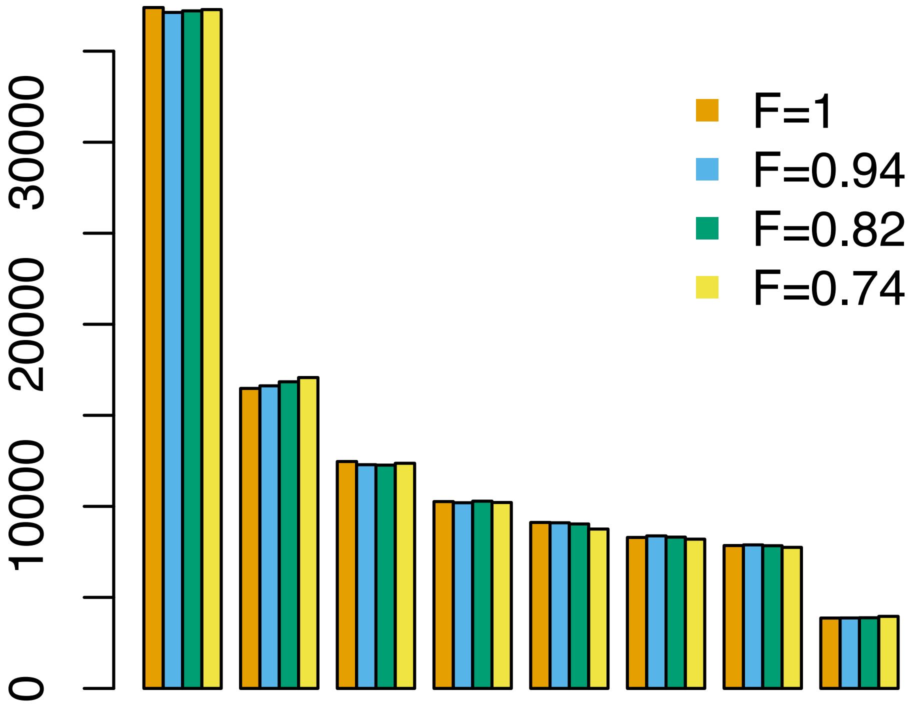

Supplementary Figure 9. Prediction of the nonsynonymous SFS under full selfing and partial selfing.

Forward simulations of the nonsynonymous SFS for *A. thaliana*, assuming either full selfing (*F*=1) or partial selfing with rates of 97% (*F*=0.94), 90% (*F*=0.82), or 85% (*F*=0.74). All four SFS agree well, suggesting that selfing in *A. thaliana* can be modeled by assuming an inbreeding coefficient of one. The simulations assume a three-epoch demographic model with parameters from Supplementary Table 1. An *h*-*s* relationship and a gamma DFE is assumed with parameters according to the genome-wide estimates using both *A. lyrata* and *A. thaliana* (Supplementary Table 4).

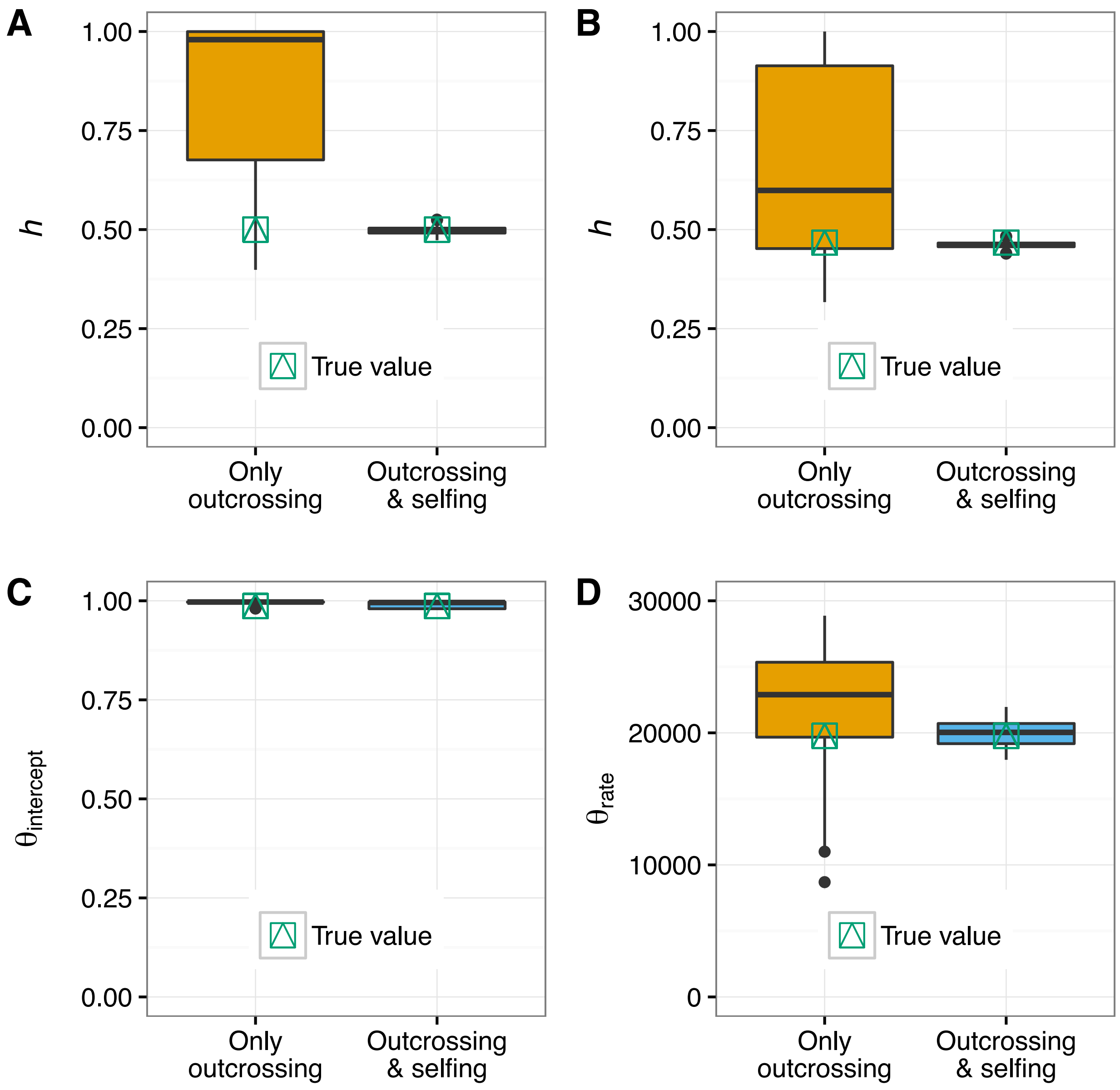

Supplementary Figure 10. Testing the inference of dominance parameters with simulations. Data are simulated under (A) an additive model (*h*=0.5), (B) a constant *h* model (*h*=0.46), and (C, D) an *h*-*s* relationship model (θ_{rate}=19773, $\theta_{intercept}$=0.986). True parameter values are indicated in green and MLEs from 100 replicates are shown as boxplots. Including the selfing species in the inference considerably improves estimation of the dominance parameters.

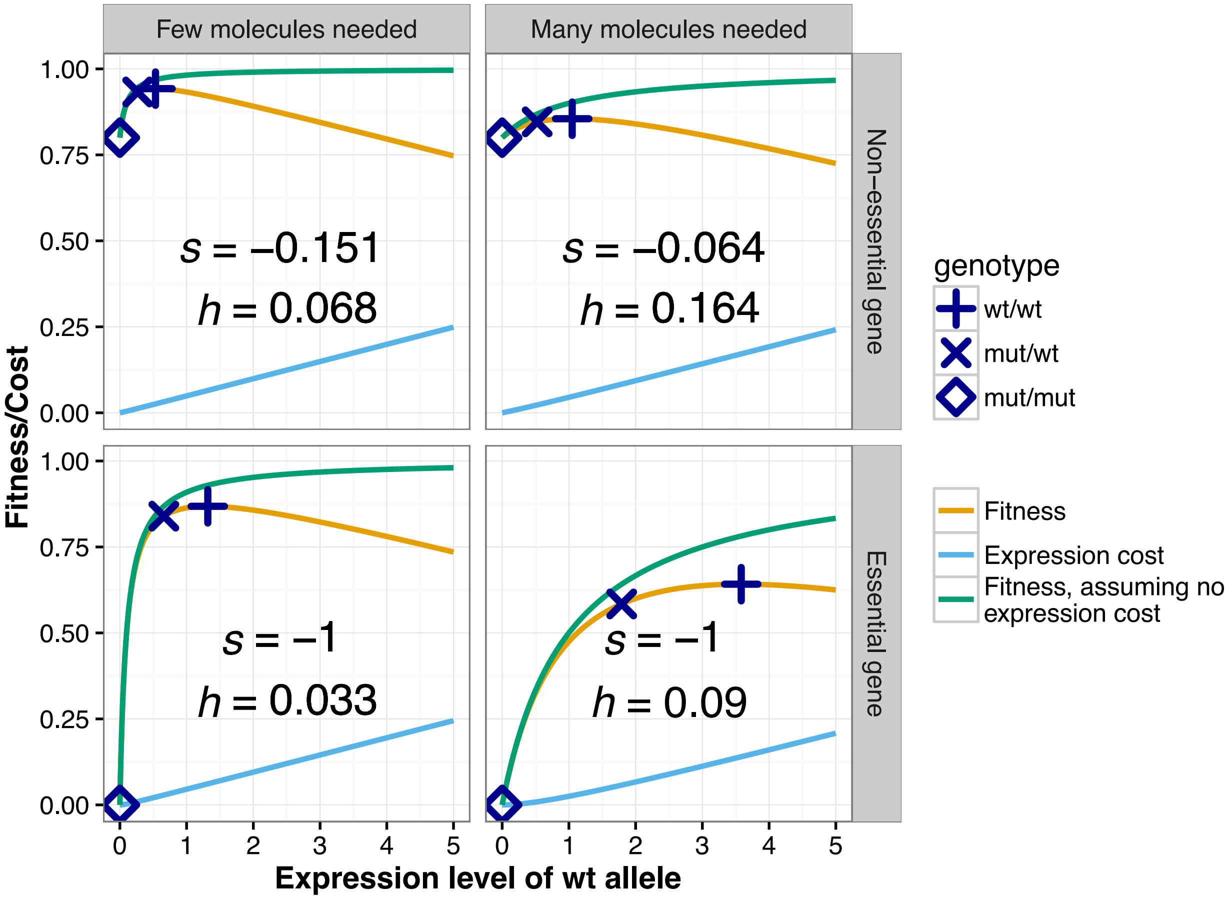

Supplementary Figure 11. Gene expression model for the evolution of dominance. Examples of the computation of selection coefficient (s) and dominance coefficient (h) under our gene expression model for the evolution of dominance. The expression level of the homozygous wild type genotype (wt/wt) maximizes fitness after taking expression cost into account. The expression level of the gene is zero when the mutant is homozygous (mt/mt), and is half the optimal expression level when the mutant is heterozygous (wt/mt). The corresponding fitness values allow computation of s and h (see Methods). For non-essential genes, s and h are negatively related, i.e. the more deleterious mutation has a smaller h value. For mutations in essential genes, the gene with the higher optimal expression level has a larger h value than the gene with the lower optimal expression level.

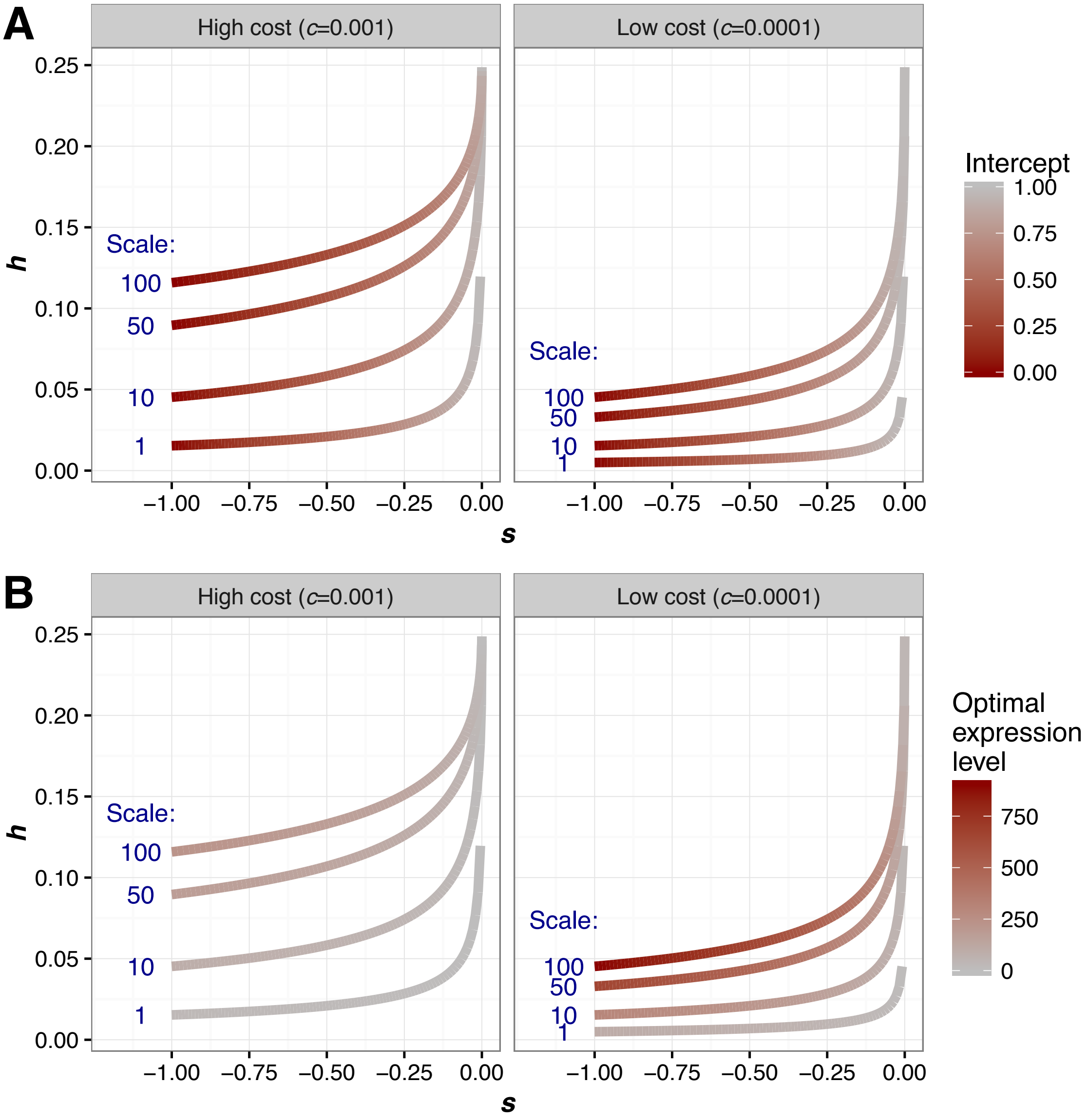

Supplementary Figure 12. Relationship between *h* and *s* under our gene expression model for the evolution of dominance.

The intercept in the model is varied continuously from 0 to 1, the scale parameter is set to 1, 10, 50, or 100, and the cost of gene expression per expression unit is set to 0.001, or 0.0001. In (A), the color scheme indicates different values of the intercept, in (B) it indicates different optimal expression levels (x_{opt}).

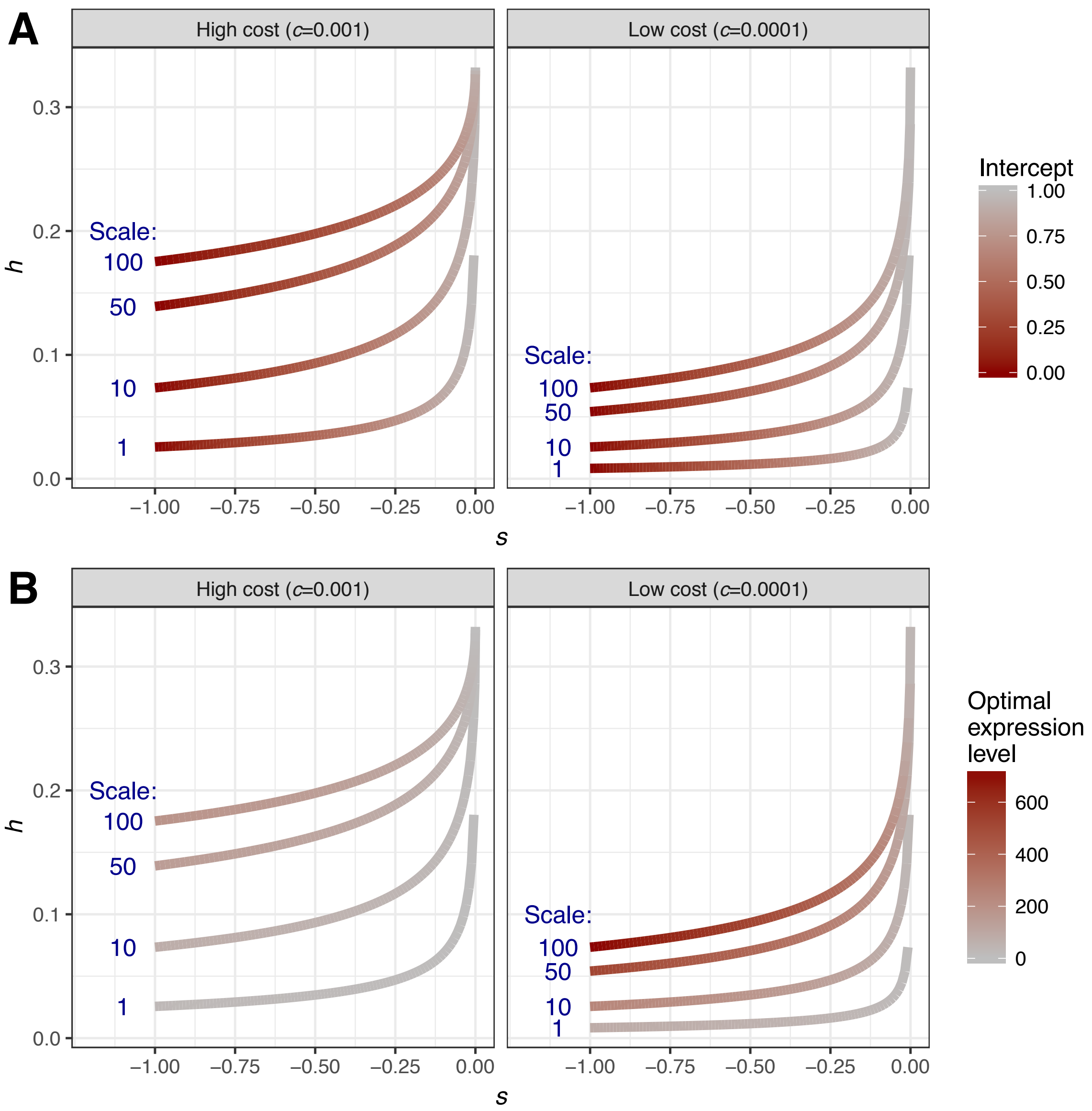

Supplementary Figure 13. Relationship between *h* and *s* under our gene expression model assuming suboptimal expression.

Our gene expression model for the evolution of dominance is modified such that the wild-type genotype has a gene expression level of only 80% of the optimal value x_{opt} (equation (5) in the main text). The intercept in the model is varied continuously from 0 to 1, the scale parameter is set to 1, 10, 50, or 100, and the cost of gene expression per expression unit is set to 0.001, or 0.0001. In (A), the color scheme indicates different values of the intercept, in (B) it indicates different optimal expression levels (x_{opt}).

Supplementary Table 1. Demographic parameter estimates.
Demographic parameter estimates for the two-epoch and the three-epoch model for *A. lyrata* and *A. thaliana*. The effective population size is indicated as N_e, *LL* is the log-likelihood, and T is the time length of the epoch in generations.

Model	Species	$N_{e,ancestral}$	$N_{e,second\ epoch}$	$N_{e,third\ epoch}$	T(second epoch)	T(third epoch)	Synonymous theta	*LL*
Two-epoch	*A. lyrata*	530,895	1,797,556	-	562,612	-	129,058	-1095
	A. thaliana	746,148	100,218	-	568,344	-	199,771	-104
Three-epoch	*A. lyrata*	608,570	6,554,858	23,584	462,952	1,489	131,613	-218
	A. thaliana	161,744	24,076	203,077	7,420	14,534	41,795	-73

Supplementary Table 2. Model comparison of dominance models.
Likelihood ratio test statistics (Λ) and *P*-values when comparing different models of dominance, using only data from *A. lyrata*. The *h-s* relationship fits the data significantly better than the additive model and significantly better than a model with a single dominance coefficient.

H0	H1	Λ	*P*-value
Additive	Constant $h \neq 0.5$	123	$<1x10^{-15}$
Additive	*h-s* relationship	492	$<1x10^{-15}$
Constant $h \neq 0.5$	*h-s* relationship	368	$<1x10^{-15}$

Supplementary Table 3. Model comparison of different *h*-*s* relationship functions. Parameter estimates and log likelihoods for different parameterizations of the DFE and the *h*-*s* relationship function. The inverse *h*-*s* relationship is defined by the formula $h=f(s)=1/((1/\theta_{intercept})-\theta_{rate}*|s|)$, the logistic relationship is defined by the formula $h=f(s)=\theta_{intercept}*(1+\exp(-\theta_{offset}))/(1+\exp(\theta_{rate}*|s|-\theta_{offset}))$.

Assumed *h* -*s* relationship	DFE	Shape	Scale	$P_{Neutral}$	$\theta_{intercept}$	θ_{rate}	θ_{offset}	*LL*
Additive	Gamma	0.24	0.0006		0.50	0.00		-914
Constant *h*	Gamma	0.24	0.0007		0.47	0.00		-885
Inverse	Gamma	0.18	0.0031		1.00	41225.56		-385
Inverse, $\theta_{intercept}$ fixed at	Gamma	0.22	0.0013		0.50	18670.56		-696
Inverse, *h* >0.5 set to 0.5	Gamma	0.22	0.0014		3.07	34493.69		-655
Inverse	Gamma & neutral point mass	0.18	0.0029	2.05E-09	1.00	36022.52		-413
Inverse, $\theta_{intercept}$ fixed at 0.5	Gamma & neutral point mass	0.22	0.0013	6.23E-08	0.50	17538.19		-698
Logistic	Gamma	0.18	0.0027		0.91	6180.87	2.45E-05	-394
Logistic $\theta_{intercept}$ fixed at 0.5	Gamma	0.21	0.0014		0.50	3579.73	1.06E-29	-672

Supplementary Table 4. Maximum likelihood estimates of DFE and dominance parameters.

Estimates for the gamma DFE parameters (shape, scale) and the two parameters of the *h-s* relationship ($\theta_{intercept}$, θ_{rate}) for different sets of genes (Genome-wide, catalytic and structural).

Gene set	Data	Inference model	Shape	Scale	$\theta_{intercept}$	θ_{rate}	*LL*
Genome-wide	Only *A. lyrata*	Additive	0.27	0.0004	0.5 (fixed)	0 (fixed)	-467
		Constant *h*	0.29	0.0002	0.999	0 (fixed)	-405
		h-s relationship	0.16	0.0092	0.978	50328	-221
	Only *A. thaliana*	Additive	0.155	0.00612	0.5 (fixed)	0 (fixed)	-84
	A. lyrata and *A. thaliana*	Additive	0.245	0.00063	0.5 (fixed)	0 (fixed)	-914
		Constant *h*	0.243	0.00073	0.467	0 (fixed)	-885
		h-s relationship	0.179	0.00311	0.998	41226	-385
Catalytic	Only *A. lyrata*	Additive	0.398	0.00017	0.5 (fixed)	0 (fixed)	-105
		Constant *h*	0.436	0.00007	0.992	0 (fixed)	-99
		h-s relationship	0.200	0.00711	0.988	50736	-74
	Only *A. thaliana*	Additive	0.158	0.01326	0.5 (fixed)	0 (fixed)	-68
	A. lyrata and *A. thaliana*	Additive	0.303	0.00048	0.5 (fixed)	0 (fixed)	-332
		Constant *h*	0.307	0.00051	0.427	0 (fixed)	-322
		h-s relationship	0.236	0.00167	1.000	46618	-203
Structural	Only *A. lyrata*	Additive	0.407	0.00062	0.5 (fixed)	0 (fixed)	-50
		Constant *h*	0.442	0.00023	0.997	0 (fixed)	-48
		h-s relationship	0.268	0.00774	0.872	30106	-43
	Only *A. thaliana*	Additive	0.272	0.00255	0.5 (fixed)	0 (fixed)	-31
	A. lyrata and *A. thaliana*	Additive	0.375	0.00082	0.5 (fixed)	0 (fixed)	-90
		Constant *h*	0.369	0.00065	0.763	0 (fixed)	-85
		h-s relationship	0.331	0.00119	0.996	18309	-80

Supplementary Table 5. Maximum likelihood estimates of DFE and dominance parameters for different expression levels and connectivity.

Estimates for the gamma DFE parameters (shape, scale) and the two parameters of the *h-s* relationship ($\theta_{intercept}$, θ_{rate}) for different sets of genes (low and high expression level, and low and high connectivity).

Gene set	Data	Inference model	Shape	Scale	$\theta_{intercept}$	θ_{rate}	*LL*
Low expression	Only *A. lyrata*	Additive	0.299	0.00020	0.5 (fixed)	0 (fixed)	-141
		Constant *h*	0.327	0.00008	0.982	0 (fixed)	-134
		h-s relationship	0.122	0.08521	0.880	93694	-97
	Only *A. thaliana*	Additive	0.132	0.00374	0.5 (fixed)	0 (fixed)	-63
	A. lyrata and *A. thaliana*	Additive	0.258	0.00030	0.5 (fixed)	0 (fixed)	-514
		Constant *h*	0.242	0.00027	0.864	0 (fixed)	-369
		h-s relationship	0.236	0.00034	0.997	35966	-328
High expression	Only *A. lyrata*	Additive	0.381	0.00033	0.5 (fixed)	0 (fixed)	-244
		Constant *h*	0.423	0.00012	0.997	0 (fixed)	-228
		h-s relationship	0.212	0.00931	0.999	36169	-177
	Only *A. thaliana*	Additive	0.186	0.01944	0.5 (fixed)	0 (fixed)	-62
	A. lyrata and *A. thaliana*	Additive	0.265	0.00183	0.5 (fixed)	0 (fixed)	-756
		Constant *h*	0.281	0.00211	0.268	0 (fixed)	-540
		h-s relationship	0.200	0.01356	0.990	33765	-264
Low connectivity	Only *A. lyrata*	Additive	0.317	0.00030	0.5 (fixed)	0 (fixed)	-151
		Constant *h*	0.349	0.00011	0.993	0 (fixed)	-143
		h-s relationship	0.177	0.00819	0.967	44449	-106
	Only *A. thaliana*	Additive	0.164	0.00508	0.5 (fixed)	0 (fixed)	-57
	A. lyrata and *A. thaliana*	Additive	0.270	0.00056	0.5 (fixed)	0 (fixed)	-320
		Constant *h*	0.268	0.00054	0.545	0 (fixed)	-316
		h-s relationship	0.222	0.00118	0.998	36278	-226
High connectivity	Only *A. lyrata*	Additive	0.361	0.00040	0.5 (fixed)	0 (fixed)	-151
		Constant *h*	0.394	0.00015	0.999	0 (fixed)	-142
		h-s relationship	0.217	0.00794	0.850	35996	-104
	Only *A. thaliana*	Additive	0.171	0.01507	0.5 (fixed)	0 (fixed)	-101
	A. lyrata and *A. thaliana*	Additive	0.295	0.00093	0.5 (fixed)	0 (fixed)	-433
		Constant *h*	0.294	0.00095	0.504	0 (fixed)	-433
		h-s relationship	0.235	0.00290	0.991	28801	-288

Supplementary Note 1

Simulated null distributions and the expectation of the asymptotic theory
The two simulated null distributions of Λ in Fig. 2c and 2d follow closely to the expectation of the asymptotic theory, with only a slightly larger mean and standard deviation: the expected mean and standard deviation of a chi-square distribution with *df*=1 is 1 and 1.9, the observed mean and standard deviation of Λ in Fig. 2c is 1.9 and 2.9. The expected mean and standard deviation of a chi-square distribution with *df*=2 is 2 and 2, the observed mean and standard deviation of Λ in Fig. 2d is 2.1 and 4.9.

Robustness of inference to model mis-specifications
When we make simultaneous use of data from both outcrossing (*A. lyrata*) and inbreeding (*A. thaliana*) species for inferring dominance, we implicitly make the assumption that the DFE is the same in both species. However, for highly diverged species such as humans and *Drosophila*, it was shown recently that the DFE, in units of *s*, is significantly different [1]. One potential concern is that differences in the DFE between species could lead to falsely inferring an *h-s* relationship when the true model is additivity.

However, we found additional support for the of *h-s* relationship model. First, we see significant support for an *h-s* relationship over an additive or constant *h* model even when basing our inference only on the outcrossing *A. lyrata* data (Supplementary Table 2). Further, the estimates of the DFE and dominance parameter estimates agree reasonably with each other across different ways of doing the inference. Specifically, estimates made using only *A. lyrata*, agree with those using *A. lyrata* and *A. thaliana* combined, although in the former case, the confidence limits are wider (Supplementary Fig. 8). Second, we explored the effect of different DFEs on our inference procedure using simulations. We ran simulations under an additive model, with parameters of the DFE taken from separate estimates of the DFE in each species (for details see Methods). Then, on each simulated dataset, we fit the demographic and selective models. Lastly, we compute the sum of log likelihoods ($LL_O + LL_I$), assuming a unique additive DFE in both species (true model). Then we compare this log likelihood to the log likelihood that assumes the same DFE, but an *h-s* relationship (incorrect model). We find that the additive log likelihood always sums up to a larger value than the log likelihood assuming the same DFE, but an *h-s* relationship. This pattern in the simulations contrasts with what is seen in the actual empirical data. For the empirical data, we find that the log likelihood of the additive model with unique DFEs ($LL_O + LL_I$ = -467 - 84 = -551) is smaller (i.e. a worse fit) than the log likelihood assuming the same DFE, but an *h-s* relationship (*LL* = -385, see Supplementary Table 4). This suggests that the additive model has a worse fit than an *h-s* relationship model, even when the assumption of an identical DFE in both species is relaxed. In summary, analyses of simulated data suggest that it is possible to distinguish between different DFEs between species and a true *h-s* relationship. It is unlikely for our inference framework to infer a spurious *h-s* relationship due to differences in the DFE between species.

Another assumption of our approach is that the inbreeding coefficient *F* of the selfing population equals 1. We tested robustness to this assumption by simulating SFS data for a selfing population with selfing rate at the lower end of what has been estimated for *A. thaliana* (97%; [2–5]). We then compared this SFS to an SFS that is simulated under full selfing (*F*=1), and found that the SFS match up well. Similar results are found for even lower selfing rates of 90% or 85% (Supplementary Fig. 9). Moreover, we found that our approach leads to unbiased estimates when

simulating data under a selfing rate of 97% (Supplementary Fig. 10). Thus, an inbreeding rate of 97% is high enough to ensure unbiased estimation of dominance parameters with our approach.

Robustness in establishing the *h*-*s* relationship

The negative relationship between h and s, such that more deleterious mutations are more recessive, was first reported by a series of mutation accumulation (MA) experiments in *Drosophila* [6,7], and later supported by two studies in yeast [8,9]. However, the validity of the results was questioned [8,10]. Further, the more comprehensive and detailed study in yeast restricts their *h*-*s* relationship models such that more deleterious mutations are only allowed to become more recessive than less deleterious mutations, but not more dominant [8]. Such a study, by definition, cannot find support for a positive relationship between h and s, because the model did not allow for such a relationship. Thus, based on previous work, it has not clearly been established that more deleterious mutations become more recessive.

Therefore, we also tested an alternative model where h converges to one instead of zero, i.e. more deleterious mutations are more dominant than less deleterious mutations:

$$h = f_{\text{alternative}}(s) = 1 - \frac{1}{\frac{1}{(1-\theta_{\text{intercept}})} - \theta_{\text{rate}} s}$$

However, this model does not improve fit to the SFS over a constant *h* model or the *h*-*s* relationship model of equation 1 in the main text. When using only data from *A. lyrata*, then the log likelihood of this alternative *h*-*s* relationship model (*LL* = -405.1) is similar to that of the constant *h* model (*LL* = -405.3), and much lower than the log likelihood of the *h*-*s* relationship model of equation 1 (*LL* = -221.1). The small estimated θ_{rate} parameter (3,980) suggests that this model is equivalent to the constant *h* model where *h* does not change with s. Similar results are obtained with our two-population inference, using data from both *A. lyrata* and *A. thaliana*. Again, the log likelihood of this alternative *h*-*s* relationship model (*LL* = -885.2) is similar to that of the constant *h* model (*LL* = -885.2), and much lower than the log likelihood of the *h*-*s* relationship model of equation 1 (*LL* = -385.1). The extremely small estimated θ_{rate} parameter (0.26) suggests that this model is equivalent to the constant *h* model. Thus, in summary, we conclude that a model where more deleterious mutations become more dominant does not fit the SFS as well as a model where more deleterious mutations become more recessive.

Supplementary References

1. Huber, C. D., Kim, B. Y., Marsden, C. D. & Lohmueller, K. E. Determining the factors driving selective effects of new nonsynonymous mutations. *Proc. Natl. Acad. Sci.* **114,** 4465–4470 (2017).
2. Platt, A. *et al.* The scale of population structure in *Arabidopsis thaliana*. *PLoS Genet.* **6,** e1000843 (2010).
3. Abbott, R. J. & Gomes, M. F. Population genetic structure and outcrossing rate of *Arabidopsis thaliana* (L.) Heynh. *Heredity* **62,** 411–418 (1989).
4. Bergelson, J., Stahl, E., Dudek, S. & Kreitman, M. Genetic variation within and among populations of *Arabidopsis thaliana*. *Genetics* **148,** 1311–1323 (1998).
5. Picó, F. X., Méndez-Vigo, B., Martínez-Zapater, J. M. & Alonso-Blanco, C. Natural genetic variation of *Arabidopsis thaliana* is geographically structured in the Iberian peninsula. *Genetics* **180,** 1009–1021 (2008).
6. Mukai, T., Chigusa, S. I., Mettler, L. E. & Crow, J. F. Mutation rate and dominance of genes affecting viability in *Drosophila melanogaster*. *Genetics* **72,** 335–355 (1972).
7. Simmons, M. J. & Crow, J. F. Mutations affecting fitness in *Drosophila* populations. *Annu. Rev. Genet.* **11,** 49–78 (1977).
8. Agrawal, A. F. & Whitlock, M. C. Inferences about the distribution of dominance drawn from yeast gene knockout data. *Genetics* **187,** 553–566 (2011).
9. Phadnis, N. & Fry, J. D. Widespread correlations between dominance and homozygous effects of mutations: implications for theories of dominance. *Genetics* **171,** 385–392 (2005).
10. García-Dorado, A. & Caballero, A. On the average coefficient of dominance of deleterious spontaneous mutations. *Genetics* **155,** 1991–2001 (2000).
